# Clinical Outcome and Fracture Risk Prediction of Benign Bone Tumors on the Acetabular Dome: 7-Year Clinical Experience and a Finite Element Analysis

**DOI:** 10.1155/2022/5150474

**Published:** 2022-03-14

**Authors:** Hongsheng Yang, Nishant Banskota, Xiang Fang, Yan Xiong, Wenli Zhang, Hong Duan

**Affiliations:** Department of Orthopedics, Orthopedic Research Institute, West China Hospital, Sichuan University, Chengdu, Sichuan, China

## Abstract

The treatment of benign pelvic lesions and tumors is still a challenge in clinical orthopedics. The surgical procedure was complicated and the postoperative complication was hard to avoid usually. The purpose of this study is to analyze the clinical outcome and predict the fracture risk of benign bone tumors on acetabular dome by finite element analysis. In our research, clinical data of 25 patients were collected from January 2010 to January 2017, including basic information of patients, reconstruction methods, complications, and postoperative MSTS function scores. Finite element analysis (FEA) was used to predict the fracture risk when a benign tumor involved an acetabular dome. 25 patients were followed up for 37.5 ± 5.6 (ranging from 24 to 78) months. Intraoperative bleeding was 100–3000 ml (mean 858.3 ml). The postoperative MSTS93 score was 19.61 ± 7.32 before operation and 26.28 ± 15.59 at the last follow-up. The results of finite element analysis suggest that there was a high risk for pathological fracture in the following: both columns were damaged by tumors; the anterior column and 50% of the posterior column were affected. Other cases were in the low fracture risk group. Based on this study, we believe that, according to the risk assessment results of tumor cavity fracture suggested by the FEA results, combined with the nature of tumor, it may become a useful tool which is a great significance to guide the operation plan, select the operation time, and guide the postoperative functional exercise.

## 1. Introduction

Bone tumors involving acetabulum have been a great challenge in diagnosis and treatment [1–3] because of their deep location, complex anatomical structure and adjacent to important organs, vessels, and nerves [4–6]. Extensive resection of tumors reconstructs the stability of pelvis and acetabulum as much as possible, so as to reduce recurrence and avoid amputation, which is a recognized treatment scheme for acetabular malignant tumors [7–9]. However, for benign bone tumors in the acetabulum, such as giant cell tumor (GCT), fibrous dysplasia (FD), chondroblastoma, bone cyst, and Langerhans cell dysplasia, there is still no unified standard in the treatment, although the Enneking system has a good classification of tumor activity [2, 6, 10–13]. Bone tumors involving the acetabular dome will affect the weight-bearing function of the acetabulum, which is very important for the stability of the hip joint. In the clinical field, most surgeons make treatment decisions based on personal experience, which makes the treatment of benign acetabular dome bone tumors lack a recognized standard. The finite element analysis (FEA) can be used as an important tool for structural mechanical analysis of orthopedics. It has a special significance in structural mechanical analysis of pelvis [14–16]. However, there have been no reports on the biomechanical analysis of benign neoplastic bone defects in the acetabulum in the past research. Therefore, this study aimed to summarize the clinical outcome of benign bone tumors on acetabular dome and predict fracture risk by established 3D finite element analysis, which provides a basis for assessing the timing of surgery, developing surgical plans, and guiding postoperative rehabilitation.

## 2. Materials and Methods

### 2.1. Part of Clinical Information

This is a retrospective study. The study obtained the informed consent of all patients and was approved by the research ethics committee of our institution.

Inclusion criteria were as follows: a benign lesion with histology-based evidence; aggressive, active lesion with growing in volume; intractable pain, gait abnormal with activity limitation of the hip joint; and large in size with a high risk of pathological fracture. Exclusion criteria were as follows: nonsurgery procedure; malignant tumor; and a pelvic tumor without involving acetabular dome.

From January 2010 to January 2017, according to the inclusion and exclusion criteria, all patients with benign bone tumors on the acetabulum dome were enrolled in this study at our institution, following different surgeries of curettage, bone graft, or resection. All of these surgeries were performed by a single surgeon team, and the postoperative check and follow-up were also completed under the supervision of the same team. The clinical data include age and gender of patients, location and size of the tumor and postoperative complications, recurrence, and function scores (Musculoskeletal Tumor Society Score-93 (MSTS-93).

The pathological diagnoses of patients were confirmed by preoperative biopsy or intraoperative frozen. Preoperative chest X-rays or chest CT were used to exclude pulmonary metastasis. The follow-up results were recorded according to the outpatient follow-up results.

### 2.2. Part of Finite Element Analysis [17]

**Figure 1:**
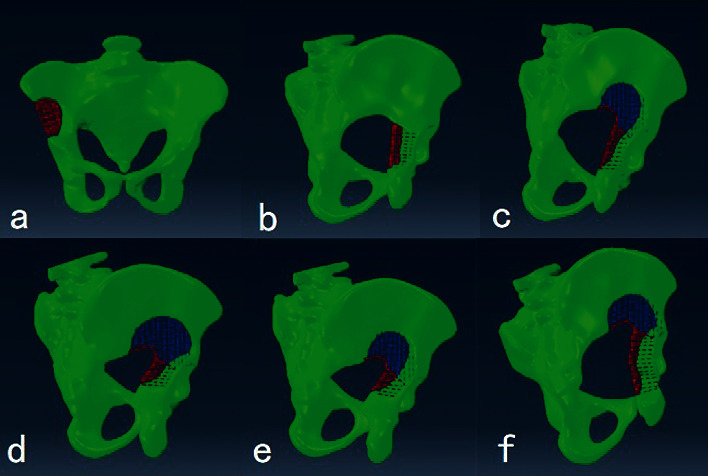
Six types of bone defects on acetabular dome models:(a) type (I), (b) type II, (c) type III, (d) type IV, (e) type (V), and (f) type VI. The figure is reproduced from Hongsheng Yang et al. 2020.

The pelvis of a normal patient (26 years old, male, 173cm height, 60kg weight) was scanned by CT (Netherlands, Philips Healthcare, Brilliance 64). The data were imported into Mimics V15.0 software in the DICOM format to reconstruct the model of pelvis and proximal femur. According to the previous research [18, 19], the tissue material parameters of the model were as follows: the elastic modulus of cortical bone was 17gpa; Poisson's ratio was 0.3; and the fatigue strength of the upper and lower segments of pubis was 150 MPa and that of acetabulum is 120 MPa. Different types of bone defect models on acetabular dome were simulated. All parts were imported into ABAQUS 6.13 for assembly and meshing, and the free mesh solid model was adopted (nodes and element number of each model are shown in Table 1). At last, we established six kinds of bone defect models 5 mm away from the acetabular dome based on the division of acetabular column by a Judet–Letournel classification system. Six bone defect models are as follows: Type I, the anterior column (resection of the anterior column above the inferior margin of the acetabulum in the pelvic AP position); Type II, posterior column (resection of the posterior column above the superior margin of the acetabulum in the pelvic AP position); Type III, 1/2 anterior column + posterior column (resection of 1/2 anterior column at the medial part of acetabulum dome and the connected posterior column above the lower edge of the acetabulum); Type IV, anterior column + 1/2 posterior column (resection of the anterior column of the acetabulum and 1/2 connected posterior column); Type V, 1/2 anterior column+ 1/2 posterior column (resection of the 1/2 anterior column and 1/2 posterior column connecting to acetabular dome); and Type VI, anterior column + posterior column (all anterior and posterior columns of the acetabulum dome were removed) (Figure 1).

Six types of bone defects on four different positions were analyzed through software including sitting position, standing position, affected one-legged standing position, and affected one-legged jumping position. In reference to previous studies [18–20], a vertical load of 500 N was applied to the upper surface of the sacral 1 vertebral body in three postures: sitting, standing, and one-legged standing position, so as to simulate the influence of the gravity of the upper body on the pelvis. When simulating the one-legged jumping position, the stress loading of the acetabular dome was 1000 N. In this study, the acetabular fatigue strength was set as 120 MPa.

### 2.3. Statistical Analysis

Continuous data are expressed as mean ± standard deviation. The normality of the continuous data was tested by the one-sample Kolmogorov–Smirnov test. Independent sample *t*-test and Mann–Whitney U were, respectively, used to check normally distributed parameters and non-normally distributed parameters. A *p*-value of 0.05 or less was considered statistically significant. Statistical analysis was performed with the use of SPSS Statistics software version 23.0 (IBM, Armonk, NY).

## 3. Results

### 3.1. Clinical Effect Analysis

A consecutive series of 25 patients (16 males and 9 females, ranging from 14 to 60 years, mean age 39.4 years) with benign bone tumors on the acetabulum dome were enrolled in this study. All observations are recorded in Table 2.

**Figure 2:**
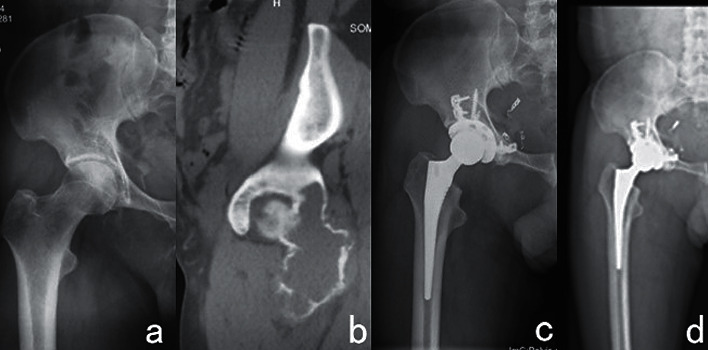
A 44-year-old man with GCT of bone in sections II and III of the right pelvis underwent tumor resection, acetabular reconstruction, total hip replacement, autogenous femoral head, acetabular bone graft, plate and screw fixation, sciatic nerve exploration, and muscle origin reconstruction. (a) Preoperative X-ray. (b) Preoperative CT scan. (c) X-ray at 6 months after the operation. (d) X-ray at 12 months after the operation.

**Figure 3:**
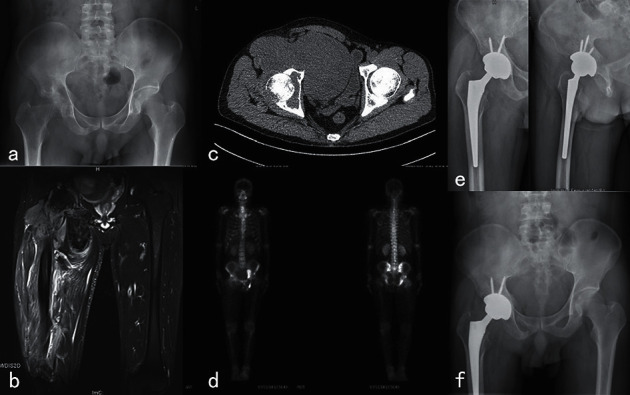
40-year-old male, giant cell tumor of the tendon sheath in sections I and II of right hemipelvis. First-stage surgery of most of tumor resection and exploration of iliac vessels and the sciatic nerve. Second-stage surgery of resection of residual tumor, total hip arthroplasty, autologous bone graft, and allograft acetabular reconstruction. (a) Preoperative X-ray, (b) preoperative CT scan, (c) preoperative MRI, (d) preoperative bone scan, (e) X-ray at 6 months after an operation on anteroposterior and lateral position, and (f) X-ray at 12 months after the operation.

Surgical treatments include the following: (1) tumor resection, total hip replacement, acetabulum reconstruction with femoral head (Figure 2); (2) tumor resection with modular hemi-pelvic replacement; and (3) curettage with bone grafting or bone cement. The thickness of the tumor cavity removed by high-speed burr during surgery was 1 mm and then the cavity was filled with 95% anhydrous alcohol for 15–20 minutes to eliminate maximum residual tumor cells. I^125^ particle implantation was used in two cases to reduce local recurrence. Local recurrence occurred at one year after surgery in one case of giant cell tumor of bone accompanied with an aneurysmal bone cyst in sections II and III of the pelvis and was treated with tumor resection. Xgeva (denosumab), calcium, and vitamin *D* were used to prevent local recurrence in one case of diffuse giant cell tumor of the tendon sheath in sections I, II, and IV of pelvis. The whole procedure was completed in two successive operations (Figure 3).

Outpatients review included physical examination, imaging evaluation, and MSTS functional scoring [21] (Table 3). Thoracic CT was performed in patients with aggressive tumors to exclude pulmonary metastasis.

The median follow-up time was 37.5 ± 5.6 months (ranging from 24 to 78 months). No distant metastasis was observed after surgery. Local recurrence was observed in one case of GCT of bone at second year after the operation and was treated with tumor extend resection surgery. Bilateral pleural effusion occurred in one patient and was treated with repeated pleural puncture fluid and respiratory rehabilitation training. Incision complications were seen in 10 patients including 5 cases of incision effusion and 5 cases of incision fat liquefaction. All 10 cases were healed without further infection.

Osseointegration of the graft bone was achieved in 13 patients who underwent curettage and bone grafting at 12–15 months after the operation and the bone strengths of the acetabular dome showed by X-ray were satisfactory. No prosthesis loosening, detachment, displacement, or rupture was observed in 8 patients after tumor resection and prosthesis replacement. The preoperative MSTS93 score was 19.61 ± 7.32. The postoperative MSTS93 score was 21.95 ± 7.38 and 25.12 ± 6.38 at three and six months after surgery. The final follow-up MSTS score was 26.28 ± 15.59 which was significantly improved than pre-operation (*P* < 0.01).

### 3.2. Finite Element Analysis

In a normal pelvic model, the maximum stress in the sitting position was 1.5 MPa, which appeared in the sciatic tuberosity. The maximum stress in the standing position was 0.6 MPa, which appeared at the superior border of the greater sciatic notch. The maximum stress in the affected one-legged standing position was 3.5  MPa, which appeared at the upper edge of the greater sciatic notch.

**Figure 4:**
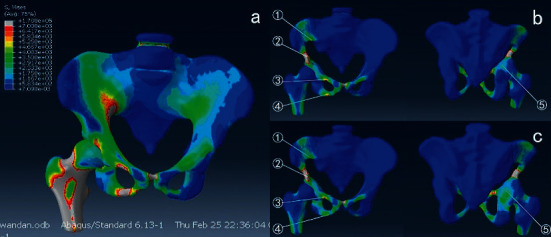
(a) stress distribution of normal pelvis in affected one-legged jumping position. (b) Stress distribution of type III bone defect model ①33.0 MPa ②106.7Mpa ③48.1 MPa ④47.4Mpa ⑤13.8Mpa. (c) ①17.8 MPa ②114Mpa ③38.9 MPa ④20.4Mpa ⑤28.9Mpa. Figure 4 is reproduced from Hongsheng Yang et al. 2020.

In six different types of bone defects, the maximum stresses were all observed in the affected one-legged jumping position [17]. In the Type I, Type II, Type III, and Type V bone defect models, the maximum stress was 22.8  MPa, 36.0  MPa, 40.6  MPa, and 30.9  MPa, respectively, which were far less than the acetabular fatigue strength of 120  MPa. In the Type IV and Type VI bone defect models, the maximum stress was 106.7  MPa and 114.0  MPa, respectively, which were close to acetabular fatigue strength with the risk of fracture (Figure 4).

## 4. Discussion

The assessment of the pathological fracture risk for tumor or tumor cavity on the acetabular dome had been difficult [9, 11, 22], which is of great significance in preoperative preparation, operation scheme formulation, and postoperative rehabilitation [23, 24]. Based on previous studies, there is no complete evaluation standard for pathological fracture of acetabular dome. For pathological fracture risk assessment of metastatic tumors, mirels score ≥8 implies a high risk of fracture and is an indication of preventive surgery [24]. One study by Damron T et al reported that the prediction of metastatic fractures was more accurate by CT-based structural stiffness analysis than that by the Mirels scoring system [23, 25]. However, there are few studies on the risk assessment of fractures caused by benign acetabular bone tumors, and there is still a lack of recognized evaluation criteria.

In this study, we established a bone defect FEA model, which was 5 mm away from the acetabular dome, to provide data of stress concentration in the benign bone defect model of acetabular dome from the perspective of biomechanics, so as to predict the risk of fracture. To our best knowledge, this has not been previously reported. The model showed that the risk of pathological fracture was higher when the lesion involved both columns or both anterior and 50% posterior columns. On the other hand, the risk of fracture is relatively low, such as a single anterior or posterior column. The lesions involved 50% of the anterior and posterior columns. The lesion involved 50% of the anterior column and 50% of the posterior column at the top of the acetabulum. We believe that the result is important for the treatment of benign bone tumors of the acetabulum dome in two aspects: (1) preoperatively, it can predict the risk of pathological fracture of the tumor and guide the timing of surgery and (2) it has an important role in guiding the development of the plan for the management of bone defects after tumor resection, and it can also guide postoperative functional exercise.

For preoperative pathological fracture evaluation, the nature of the tumor is an important consideration for surgery, which is classified by the Enneking classification [26]. Based on the results of FEA and the staging of the tumor, we believe that benign bone tumor lesions on the acetabulum dome can be classified into three types: (a) the low fracture risk noninvasive lesion group (static or active lesion, Enneking 1 and 2), (b) the low fracture risk invasive lesion group (Enneking 3), and (c) the high fracture risk group.

According to this division, patients with low fracture risk noninvasive lesions may achieve satisfactory results with nonoperative treatment because the lesions are limited in extent and have minimal impact on the stability of the acetabular dome, whereas those who had low fracture risk invasive lesions may need surgical treatment with appropriate adjuvant therapy when needed. It can be suggested that patients continue normal activities after diagnosis and operators have sufficient time to arrange appropriate adjuvant treatment and preoperative preparation, so as to reduce recurrence and difficulty of surgery [27–29]. For patients in the high-risk fracture group, regardless of the nature of the tumor, the operation should be carried out as soon as possible to reduce the risk of pathological fracture, the tumor recurrence, and the difficulty [30–33].

In this study, patients were not grouped for treatment according to the above criteria for the following reasons: the physicians in our study center did not have enough experience in the early evaluation of bone tumors of the acetabulum dome, less invasive biopsy operations were not performed preoperatively, and the nature of tumor could not be determined. In order to prevent the tumor spreading, some patients with the low fracture risk noninvasive lesion were operated. We have avoided these situations in the clinical studies conducted after the development of this standard, and the results will be presented in a separate study.

Second, we believe that the fracture risk assessment of bone defect after resection of acetabular dome tumor is of great significance to guide the surgery plan, as well as the way of postoperative functional exercise. For patients who can be divided into the low fracture risk group after tumor resection, the doctor has a wider choice of graft material or filling reconstruction in the bone defect when taking the operation plan. Even if bone grafting alone was conducted, with or without internal fixation, they can walk down early, which will reduce the difficulty of the operation and is conducive to the saving of resources and costs. It is also conducive to the functional recovery of patients. However, for patients in the high-risk group after tumor resection, doctors should adopt solid treatment methods for tumor defects. If it is impossible to fill with bone cement, add internal fixation, or reconstruct the pelvic defect area during operation, patients should be less active and give priority to non-weight exercise after surgery, until strength is better restored in the defect area, to reduce the risk of refracture and internal fixation failure [28, 30, 33–36].

25 patients with benign bone tumors of the acetabulum dome were treated in our institution over 7-year and 3 main ways. We are used to reconstructing the tumorigenic bone defect, including total hip replacement and acetabulum reconstruction, bone grafting or bone cement, and modular hemi-pelvic replacement, which are the main methods for tumor bone defects of acetabulum dome [14, 29, 35, 36]. However, the development of customized prosthesis or spacer produced by 3D printing technology provides a new choice for the reconstruction of tumor bone defects in this area. According to the reports, personalized 3D printed prosthesis was reported to improve individual matching and functional results and will be the future development direction [33, 37–39].

The local recurrence of acetabular dome tumor is closely related to the pathological grade, location, surgery edge, reconstruction method, adjuvant treatment, etc. [27, 29, 32]. In this study, one case of GCT recurred one year after the operation and was cured after extensive resection again. GCT of bone has certain invasiveness and is easy to relapse after the local operation, with a recurrence rate of 20%–50% [40, 41]. Guo et al. [40] believed that the tumor recurrence rate of giant cell tumor of bone increased by breaking through the envelope and local curettage, especially simple curettage in the focus; the recurrence rate can be significantly reduced by giving active treatment to the curettage of the cyst wall, including high-speed grinding drill to grind off the bone cyst wall, argon knife cauterization, and tumor segment resection. It has been reported that bisphosphonates may reduce the recurrence of giant cell tumors of bone [42]. A total of 40.0% (10/25) of the cases in this group had wound complications, all of which were delayed wound healing during the perioperative period, and no deep infection occurred. The incision problems were effectively controlled by adequate drainage, elimination of dead cavity, antibiotics, dressing change, etc.; massive hemorrhage is a common complication of pelvic tumor surgery, with a maximum of 15000 ml [27, 40, 41]. The intraoperative bleeding in this study was 100–3000 ml, with an average of 853.3 ml. Bleeding is mainly caused by tumor stripping, exposure, and resection. If the reconstruction is complex and the operation time is long, bleeding will be increased. For invasive tumors, careful preoperative planning should be performed, which includes interventional embolization, intraoperative balloon occlusion of the abdominal aorta, appropriate surgical approach, careful dissection, and appropriate reconstruction. At the same time, the tumor should be completely removed to reduce surgical complications.

### 4.1. Limitations

In this study, the FEA model did not simulate and reconstruct the cancellous bone and pelvic muscle due to the lack of micro CT and further computer hardware support. In addition, people's posture and gait have a great impact on the pelvic load-bearing function, which can be close to 10 times that of static state when running and jumping. Therefore, the stress analysis of our bone defect model cannot predict the risk of pathological fracture in all states. In addition, the reduction of pelvic bone strength due to age is also a high risk factor for pathological fracture, which may make this study underestimate the risk of fracture.

Limited by the number of samples, it leads to selection bias, which limits generalizability of the results. Larger sample sizes might have led to more significant univariate associations. The study did not consider the impact of tumor nature itself on bone quality, which has a certain impact on the results [7, 17, 34]. Limited by the follow-up time, the long-term changes of the lesions were not known. All these limitations were important factors that might have led to bias in this study.

## 5. Conclusion

Benign bone tumors involving the acetabulum dome have any postoperative complications. Good clinical results can be achieved by making a careful preoperative plan and actively dealing with postoperative complications. Groups can be divided according to the results of pathological fracture risk and the nature of the tumor, which is of great significance to guide the operation plan, select the operation time, and guide the postoperative functional exercise.

## Figures and Tables

**Table 1:** Pelvic model parameters.

Model	Type I	Type II	Type III	Type IV	Type V	Type VI	Normal pelvic
Units	205277	210649	206170	205739	208338	204499	204506
Nodes	57205	58512	57613	57522	58051	57256	56913
Minimum unit volume (mm3)	0.0003284	0.0003284	0.0003056	0.0003031	0.0004396	0.0002472	0.0004418
Maximum unit volume （mm3）	780	1006	1090	1211	1075	1055	829
Total volume（mm3）	120604	125509	118827	118006	120459	115890	127133

**Table 2:** Patients' diagnoses and operative data. General and follow-up data of patients.

Case number	Gender/Age	Diagnosis/Enneking grade	Site	Surgical resection Type	Size (cm^3^)	Operative type	Blood loss (mL)	Complication
1	M/44	GCT/3	P Column	II, III	8 × 8 × 10	Tumor resection, total hip arthroplasty, and acetabular reconstruction with autologous femoral head	2700	Incision fat liquefaction
2	F/50	GCT with ABC/3	P Column	II, III	5 × 7 × 9	Tumor resection, modular hemipelvic replacement	2500	Incision effusion
3	M/16	FB/1	A + P column	I, II	5.5 × 11.6 × 10.4	Curettage, artificial bone graft	2200	None
4	F/45	FB/1	A + P column	I, II	4.5 × 5 × 5.5	Curettage, iliac bone, artificial bone graft	400	Incision effusion
5	F/14	Hemangioma/2	A column	I, II	3 × 3 × 4	Curettage, allograft, artificial bone graft	400	None
6	M/25	ABC/2	A column	II	2.4 × 3 × 3.8	Curettage, allograft, artificial bone graft	200	None
7	M/22	Langerhans cell histiocytosis/2	A column	I, II	2.2 × 2.7 × 3.7	Curettage, artificial bone graft	250	None
8	M/41	GCT with ABC/3	A + P column, Sacroiliac joint	I, IV	11.9 × 13.1 × 14.5	Tumor resection, stability reconstruction with autologous bone, and internal fixation	3000	Incision effusion
9	M/31	FB/1	A + P column	I, II	8 × 10 × 12.5	Curettage, iliac bone, artificial bone graft	500	None
10	F/51	Diffuse giant cell tumor of tendon sheath/3	A + P column	II, III,V	9 × 12 × 16	Tumor resection, modular hemi-pelvic replacement	2000	Incision fat liquefaction
11	M/49	Hemangioma/2	A + P column	I	4.6 × 5.6 × 7.9	Curettage, bone cement	150	None
12	F/18	Chondroblastoma/2	A + P column	I, II	7 × 8 × 11	Curettage, allograft, artificial bone graft	800	None
13	M/60	FB/1	A column	I	5.5 × 7.2 × 10.3	Curettage, iliac bone, artificial bone graft	400	None
14	F/40	ABC/3	A + P column, Sacroiliac joint	I, IV	10.1 × 13.5 × 17.3	Tumor resection, modular hemipelvic replacement	1000	Incision effusion
15	M/40	Diffuse giant cell tumor of tendon sheath/3	A + P column	I, II, V	17.5 × 20.3 × 22	Tumor resection, total hip arthroplasty, and acetabular reconstruction with autologous femoral head	500	Incision fat liquefaction
16	F/34	Hemangioma/2	Sacroiliac joint	I	2 × 2.5 × 3	Curettage, iliac bone, artificial bone graft	200	None
17	M/44	FB/1	A + P column	I, II	2.6 × 3 × 4.2	Curettage, iliac bone, artificial bone graft	100	Incision fat liquefaction
18	F/42	FB/1	P Column	II	2 × 2 × 2.1	Curettage, iliac bone, artificial bone graft	100	None
19	M/44	SBC/1	A column	II	1.5 × 1.5 × 2	Curettage, iliac bone, artificial bone graft	100	None
20	M/38	GCT with ABC//3	P Column	II, III	5 × 6.3 × 8.2	Tumor resection, total hip arthroplasty and acetabular reconstruction with autologous femoral head	2500/400	Incision effusion
21	M/32	SBC/1	A column	II	2.5 × 4.5 × 2	Curettage, iliac bone, artificial bone graft	200	None
22	M/45	FB/1	A + P column	I, II	6 × 8 × 9.5	Curettage, iliac bone, artificial bone graft	500	None
23	F/42	Hemangioma/3	Sacroiliac joint	I	2 × 2.5 × 3	Curettage, iliac bone, artificial bone graft	200	None
24	M/47	GCT with ABC/3	P Column	II, III	6.0 × 5.5 × 9	Tumor resection, modular hemi-pelvic replacement	2000	Incision fat liquefaction
25	M/54	FB/1	A column	I	5.0 × 7.5 × 8.3	Curettage, iliac bone, artificial bone graft	200	None

A column = anterior column, P column = posterior column, A + P column = anterior + posterior column, GCT = giant cell tumor ABC = aneurysmal bone cyst, SBC = simple bone cyst, FB = fibrous dysplasia.

**Table 3:** Follow-up data of the patients

Patient number	MSTS93 score				Total follow-up time (M)
	Preoperative	3 months after the operation	6 months after the operation	Last follow-up	
**1**	15	23	28	27	24
**2**	12	14	24	23	26
**3**	21	26	28	27	38
**4**	21	25	27	29	28
**5**	17	22	28	28	40
**6**	19	19	28	27	78
**7**	22	27	29	29	28
**8**	9	14	21	25	38
**9**	30	28	29	30	29
**10**	5	3	8	9	38
**11**	12	21	26	27	57
**12**	10	20	18	24	44
**13**	30	27	28	28	41
**14**	19	6	10	12	24
**15**	18	21	25	28	35
**16**	25	28	28	28	72
**17**	30	28	30	30	26
**18**	26	28-	30-	30	26
**19**	24	28	30	30	24
**20**	20	24	26	25	46
**21**	22	28	29	30	29
**22**	12	21	26	27	57
**23**	24	28	30	30	24
**24**	6	18	18	24	38
**25**	30	28	29	30	29

## Data Availability

All the summarized and analyzed data during this study are included in this published article; the original data in this study are available from the corresponding author upon reasonable request.

## Abbreviations:

FEA: Finite element analysis
MSTS-93: Musculoskeletal Tumor Society Score 93
CT: Computerized X-ray tomography
GCT: Giant cell tumor
MRI: Magnetic resonance imaging.
